# Environmental pollution in North-Eastern Italy and its influence on chronic obstructive pulmonary disease: time series modelling and analysis using visibility graphs

**DOI:** 10.1007/s11869-023-01310-7

**Published:** 2023-01-25

**Authors:** Alejandra Aranburu-Imatz, Jorge E. Jiménez-Hornero, Ignacio Morales-Cané, Pablo Jesús López-Soto

**Affiliations:** 1 Department of Nursing, Maimonides Biomedical Research Institute of Cordoba (IMIBIC), Av. Menéndez Pidal S/N., 14004 Córdoba, Spain; 2 Department of Nursing, Pharmacology and Physiotherapy, University of Cordoba, Córdoba, Spain; 3 Outpatient Clinic, Hospital Giovanni Paolo II, ULSS1 Dolomiti, Veneto, Italy; 4 Department of Electrical Engineering and Automation, Universidad de Córdoba, Córdoba, Spain; 5 Department of Nursing, Hospital Universitario Reina Sofía de Córdoba, Córdoba, Spain

**Keywords:** Respiratory disease, Chronic obstructive pulmonary disease, Hospital admissions, Italy, Time series modelling, Visibility graphs

## Abstract

The impact on human health from environmental pollution is receiving increasing attention. In the case of respiratory diseases such as chronic obstructive pulmonary disease (COPD), the relationship is now well documented. However, few studies have been carried out in areas with low population density and low industrial production, such as the province of Belluno (North-Eastern Italy). The aim of the study was to analyze the effect of exposure to certain pollutants on the temporal dynamics of hospital admissions for COPD in the province of Belluno. Daily air pollution concentration, humidity, precipitations, and temperature were collected from the air monitoring stations in Belluno. Generalized additive mixed models (GAMM) and visibility graphs were used to determine the effects of the short-term exposure to environmental agents on hospital admissions associated to COPD. In the case of the city of Belluno, the GAMM showed that hospital admissions were associated with NO_2_, PM_10_, date, and temperature, while for the city of Feltre, GAMM produced no associated variables. Several visibility graph indices (average edge overlap and interlayer mutual information) showed a significant overlap between environmental agents and hospital admission for both cities. Our study has shown that visibility graphs can be useful in establishing associations between environmental agents and COPD hospitalization in sparsely populated areas.

## Introduction

According to the World Health Organization (WHO 2018), chronic diseases were the cause of 71% of deaths in the world in 2018 in ages ranging between 30 and 70. The chronic obstructive pulmonary disease (COPD) is the third leading cause of death worldwide, causing 3.23 million deaths in 2019 (WHO 2021a). COPD is defined as irreversible airflow obstruction due to increased resistance in the airways, and it is an incurable chronic disease. However, with proper treatment, the symptoms can be alleviated, the risk of death reduced, and quality of life improved (Mosquera Pestaña 1997). The main risk factor is smoking (Wheaton et al. 2019), although other factors exist, such as indoor air pollution (Kamal et al. 2021), external air pollution (Doiron et al. 2019), or chemicals used in the work environment (Van der Molen et al. 2018; Olloquequi et al. 2018).

Over recent years, studies analyzing the relationship between environmental pollution and health have increased, especially linking long-term exposure to pollution with respiratory diseases such as asthma or COPD and cardiovascular diseases (Dominski et al. 2021; Ab Manan et al. 2018; Park et al. 2021). Environmental pollution has various sources, both anthropogenic and natural (EEA 2021a). The World Health Organization’s (WHO) Global Air Quality guidelines classify the polluting substances contained in the air into particulate matter (PM), ozone (O_3_), nitrogen dioxide (NO_2_), and sulfur dioxide (SO_2_) (WHO global air quality guidelines 2021b). According to the European Environment Agency, particulate matter (PM_2,5_) is one of the main causes of premature death and health problems in Europe (EEA 2021b). Several studies carried out in Italy over the last few years have analyzed the relationship between air pollution levels and hospital admissions due to different diseases (Gandini et al. 2018; Renzi et al 2022). These studies have shown how long- and short-term exposure to air pollutants, even in low levels, has an impact on the number of hospital admissions for respiratory diseases, with an increased risk in older people, those with low economic incomes, smokers, or those with unhealthy working conditions. It has been demonstrated how the peaks of particulate contamination levels match the spikes in hospitalizations (Paolocci et al. 2020; Pini et al. 2021; De Marco et al. 2018) and a change in PM_2,5_ concentrations directly impacts on forced expiratory volume in 1 s (FEV_1_), forced vital capacity (FVC), and forced expiratory flow 25–75% (FEF_25-75_) levels. Indeed, long-term exposure to low-level air pollution, even below the current EU or US limit values, is associated with the development of COPD (Cohen et al 2017; Pozzer et al. 2019; Zhang et al. 2021a, b; Bo et al. 2021; Raji et al. 2020; Lu et al. 2021; Shin et al. 2021; Yan et al. 2021; Han et al. 2020; Wang et al 2021; Doneva et al 2019). A correlation between contamination concentration peaks and the increase in hospitalization rates after a short period of time has also been found using different systems of analysis (Zhang et al. 2021a, b; Zhang et al. 2019).

On the basis of the above, it is of interest to carry out further studies using time series analysis of selected variables, looking for dynamic pattern correspondences or correlations between them (e.g., between hospitalization admissions derived from certain diseases and atmospheric concentrations of certain contaminants). Among others, one of the recent approaches that has been used to conduct such analysis is the Visibility Graph (VG), introduced by Lacasa (2008), which relies on the transformation of the original time series into a complex network, in this case an undirected graph, which can be either weighted or unweighted. As such, it can be considered a nonlinear characteristic model of a dynamical system represented by its time series. The main advantages of this technique can be summarized as follows: (a) It is unique, unambiguous, and easy to obtain transformations; (b) a VG gathers the core features of the original associated time series into its network topology (Lacasa and Toral 2010); and (c) VGs obtained from time series of several variables can be used to find correlations between them through Multiplex Visibility Graphs (MVG) (Carmona-Cabezas et al. 2020a).

The usefulness of this approach has been demonstrated in fields as diverse as economics (Bianchi et al. 2017), the study of earthquakes (Telesca et al. 2014) and hurricanes (Elsner et al. 2009), the analysis of propagation of computer viruses, contaminant dynamics (Carmona-Cabezas et al. 2020b; Plocoste et al. 2021) or climatology and fluid dynamics, as well as in the healthcare field, where works assessing brain dysfunctions can be found which use electroencephalographic (EEG) time series (Bhaduri and Ghosh 2015). It has also been used in the early detection of sudden cardiac arrests (Nilanjana et al. 2016), epilepsy detection from electrical characteristics of EEG signals (Hao et al. 2016; Supriya et al. 2016), the assessment of psychiatric disorders by analysis of brain activity using functional Magnetic Resonance Imaging (fMRI) data (Sannino et al. 2017), establishing the relation between the intracranial pressure (ICP) and the heart rate (HR) using MVG (Dimitri et al. 2017) and the analysis of multi-omics time series which can be used in precision medicine or to monitor health events Zheng et al. 2021), among others.

In this context, our main aim was to analyze the influence of environmental conditions and concentrations of certain pollutants on the temporal dynamics of hospital admissions for COPD in the province of Belluno (North-Eastern Italy).

## Materials and methods

### Study area

**Fig. 1:**
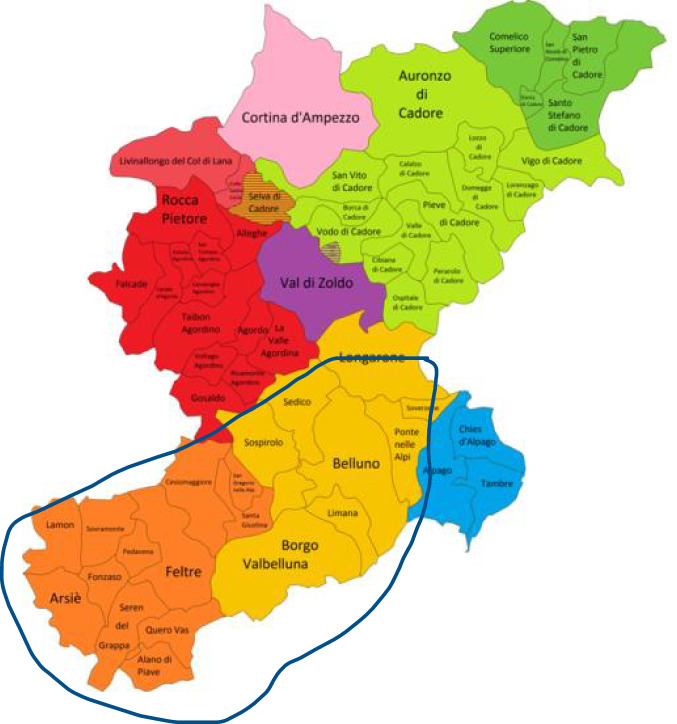
Province of Belluno and area analyzed

The province of Belluno lies in the northeast of Italy, in the Region of Veneto**.** It is the most sparsely populated province of Veneto Region, with 199,704 inhabitants and a low population density (56 people/km^2^). Located in the heart of the Dolomite Alps, Belluno is bordered to the north by Austria, to the east by the Autonomous Region of Friuli Venezia Giulia, to the west by the Autonomous Region of Trentino Alto Adige and to the south by the province of Treviso and Vicenza, which is part of the Veneto Region. The province of Belluno (Fig. 1) has unique climate conditions due to its orographic characteristics. The area covered in the present work is the valley floor of Feltre and Belluno. The climate is rather mild, with an average annual temperature between 10 and 12.5 °C. In winter, the minimum temperatures regularly go below 0 °C in January and February. In summer, the average maximum range is between 24 and 28 °C. Precipitation is rather scarce in winter and more abundant in summer with annual accumulations reaching 1600 − 1800 mm and monthly maximums between October and November of 200 − 250 mm. In the province of Belluno, the most densely inhabited areas are Belluno and Feltre. Belluno, the provincial capital, has 35,522 inhabitants, and Feltre 20,491 inhabitants (ISTAT 2022). The area we have focused on in this study starts at Ponte Nelle Alpi and ends at Quero-Vas, consisting of a long, wide valley through the Piave watercourse.

### Data

The data analyzed were the records of hospital admissions with the following characteristics: (i) patients admitted to Belluno and Feltre hospitals through the emergency department and then admitted to the medicine and pneumology units; (ii) population older than 45 years old; (iii) patients diagnosed with COPD; and (iv) in the period June 2017 − November 2019. The data were obtained from the ULSS1 Dolomiti database (belonging to the public healthcare company in Belluno province) Azienda ULSS1 Dolomiti (2022), Centro Meteo (2022). The records contained the date of birth, age, gender, residential address, date of admission and discharge, and primary discharge diagnosis. The criteria for data extraction followed the diagnosis code [ICD-9-CM 491 (International Classification of Diseases)]. The meteorological (temperature and relative humidity) and air pollutant data variables requested were obtained from the ARPAV (Regional Agency for Environmental Prevention and Protection of the Veneto) Agenzia Regionale per la Prevenzione e Protezione Ambientale del Veneto (2022). According to ARPAV, as far as air quality is concerned, any concentration data below the quantification limits have been replaced with a value equal to half of the limit itself, in coherence with the conventions used by ARPAV for the calculation of the indicators provided for by the regulation. As previously mentioned, the province has three fixed measurement stations suitable for data collection: Belluno station, Feltre station, and Alpago station. These meteorological stations are classified into different categories (traffic, industrial, and background) as well as by the location zone (urban, suburban, or rural), following the criteria by (Larssen et al.^1999^). Belluno and Feltre stations are located in urban residential areas, where the main sources of emissions are traffic and combustion from residential sectors. Pollutant variables measured from both stations are shown in Table 1.

**Table 1:** Pollutants measured at each station

	PM _2,5 µg/m_^3^	PM _10 µg/m_^3^	NO _µg/m_^3^	NO_2 µg/m_^3^	NO_X µg/m_^3^	SO_2 µg/m_^3^	O_3 µg/m_^3^
Belluno station		X	X	X	X	X	X
Feltre station	X	X	X	X	X		X

### Time series modelling and analysis

#### Generalized additive mixed models

To analyze the time series quantitative data, we used descriptive statistics with measures of frequency, central tendency, and dispersion. Normality and homogeneity were calculated using the Shapiro–Wilk and Levene tests. Comparisons for continuous variables were assessed by *t*-test, and the categorical data were compared using χ^2^ test. For the comparison of mean values between continuous variables, we used a one-factor ANOVA test and for correlation between quantitative variables, Spearman rank correlation. In order to capture the non-linear time effects on the response variable (hospital admissions), we performed two Generalized additive mixed models (GAMM), one per study area (Belluno and Feltre). All hypothesis tests were bilateral. All tests with a confidence level of 95% (*p* < 0.05) were considered statistically significant.

#### Visibility graphs

A visibility graph (VG) is a mathematical entity constituted by nodes, which correspond to the points of the transformed time series, and the edges that connect them. Two nodes at times ${t}_{a}$ and ${t}_{b}$ with values of the analyzed variable ${y}_{a}$ and ${y}_{b}$, respectively, are linked if any intermediate node at ${t}_{c}$ between them (${t}_{a}$ < ${t}_{c}$ < ${t}_{b}$) and intensity ${y}_{c}$ fulfils Eq. (1) (visibility criterion). Two adjacent-in-time nodes are always connected because there are no intermediate nodes between them.

1 $${y}_{c}<{y}_{a}+\left({y}_{b}-{y}_{a}\right)\frac{{t}_{c}-{t}_{a}}{{t}_{b}-{t}_{a}}$$

The criterion from Eq. (1) corresponds to the so-called natural visibility graph (NVG); however, it is possible to apply other visibility criteria to transform the time series, for example, the one used to construct the horizontal visibility graph (HVG) (Luque et al. 2009), which is simpler to obtain, although there is some information loss from the original time series.

The application of visibility criterion (1) leads to a symmetric square $NxN$ sparse binary adjacency matrix (2) ($N$ being the number of nodes) representative of the VG, where each row contains the information associated to each node, so that ${a}_{ij}=1$ means that nodes $i$ and $j$ have visibility (an edge connects them) and ${a}_{ij}=0$ means no visibility. Additionally, the diagonal elements of this matrix are always zero (hollow matrix), because a node has no visibility with itself and the elements surrounding the diagonal are equal to 1, because a node always has visibility with its adjacent nodes.

2 $$\left(\begin{array}{cccc}0& 1& \dots & {a}_{1N}\\ 1& 0& 1& \dots \\ \dots & 1& \dots & 1\\ {a}_{N1}& \dots & 1& 0\end{array}\right)$$

Several centrality measurements or properties of the adjacency matrix can be defined, among which the degree of each node (${k}_{i}$) can be highlighted, which counts the number of nodes connected to it through an edge using the visibility criterion (${k}_{i}=\sum_{j}{a}_{ij}$). This value is an indication of the relative importance of a node, since the nodes with the highest degrees are normally those with the highest values of the analyzed variable in the time series too. One important characteristic of the VG obtained from the degrees is the degree probability distribution $P\left(k\right)$, which is calculated for each degree by dividing the number of nodes with that degree by the total number of nodes. It is known that $P\left(k\right)$ can provide information about the nature of the original time series, for example, its periodic, chaotic, random, or multifractal behavior (Lacasa et al. 2008; Mali et al. 2018).

Multivariate analysis can be carried out using the multiplex visibility graph (MVG) approach, based on multi-layered networks, each of which is constituted by the VG of the $M$ involved time series. Therefore, an MVG is represented by the vector of adjacency matrices of the constituent VGs, i.e., $\Omega =\left\{{A}^{\left[1\right]},{A}^{\left[2\right]}, \dots ,{A}^{\left[M\right]}\right\}$, being ${A}^{\left[\alpha \right]}$ the adjacency matrix of the VG corresponding to the time series of the $\alpha$ variable (layer) and ${a}_{ij}^{\left[\alpha \right]}$ the $ij$-element of that matrix ${A}^{\left[\alpha \right]}$. Two measurements obtained from a MVG are mainly used to perform multivariate analysis (Lacasa et al. 2015; Nicosia and Latora 2015): average edge overlap ($\omega$) and interlayer mutual information ($IM$). The former quantifies, on average, the degree of overlap of the edges between any pair of nodes across the different VGs in the MVG (3), while the latter determines the correlation of the degree probability distributions $P\left({k}^{\left[\alpha \right]}\right)$ and $P\left({k}^{\left[\beta \right]}\right)$ of the two VGs corresponding to layers $\alpha$ and $\beta$ of the MVG (4).

3 $$\omega =\frac{\sum_{i}\sum_{j>i}\sum_{\alpha }{a}_{ij}^{\left[\alpha \right]}}{M\cdot \sum_{i}\sum_{j>i}\left(1-{\delta }_{0,\sum_{\alpha }{a}_{ij}^{\left[\alpha \right]}}\right)}$$

${\delta }_{0,\sum_{\alpha }{a}_{ij}^{\left[\alpha \right]}}$ is the Kronecker delta, which is equal to 1 if $\sum_{\alpha }{a}_{ij}^{\left[\alpha \right]}$ is null and 0 otherwise. $\omega$ has 1 as maximum value, meaning that the time profile of the analyzed series are identical, and $1/M$ as its minimum value, which means that every edge in the MVG only exists in one layer; therefore, a high value of $\omega$ (close to 1) indicates a high correlation of the time series involved.

4 $$IM=\sum_{{k}^{\left[\alpha \right]}}\sum_{{k}^{\left[\beta \right]}}P\left({k}^{\left[\alpha \right]},{k}^{\left[\beta \right]}\right)log\frac{P\left({k}^{\left[\alpha \right]},{k}^{\left[\beta \right]}\right)}{P\left({k}^{\left[\alpha \right]}\right)\cdot P\left({k}^{\left[\beta \right]}\right)}$$

$P\left({k}^{\left[\alpha \right]},{k}^{\left[\beta \right]}\right)$ is the joint probability distribution of having degree ${k}^{\left[\alpha \right]}$ in layer $\alpha$ and degree ${k}^{\left[\beta \right]}$ in layer $\beta$ (5).

5 $$P\left({k}^{\left[\alpha \right]},{k}^{\left[\beta \right]}\right)=\frac{{N}_{{k}^{\left[\alpha \right]},{k}^{\left[\beta \right]}}}{N}$$

where ${N}_{{k}^{\left[\alpha \right]},{k}^{\left[\beta \right]}}$ is the number of nodes at the same time instant which have degree ${k}^{\left[\alpha \right]}$ in layer $\alpha$ and degree ${k}^{\left[\beta \right]}$ in layer $\beta$. There is no theoretical upper limit for IM, but the higher it is around 1, the higher is the correlation between the degree probability distributions $P\left({k}^{\left[\alpha \right]}\right)$ and $P\left({k}^{\left[\beta \right]}\right)$.

## Results

A total of 745 COPD hospital admissions were derived from the emergency department, while other types of admissions were recorded in the hospitals of Belluno and Feltre, from June 2017 to November 2019. Table 2 shows the average values, standard deviation, and range of length of hospital stay and their distribution according to area, age and sex.

**Table 2:** General characteristics of total hospital admissions for COPD from June 2017 to May 2019. ULSS 1 Dolomiti, Italy

	Variables	Total admissions	Length of hospital stay (days)	
			Mean (SD)	Min–max
Feltre area		**300**		
	Age			
	Adults (45 − 65)	42	5.67 (2.31)	(3–7)
	Elderly (> 65)	258	10.09 (7.77)	(1 − 39)
	Sex			
	Men	149	8.86 (5.81)	(1 − 26)
	Women	151	12.75 (11.90)	(2 − 39)
	Access structure			
	First aid Feltre	264	9 (5.57)	(3 − 26)
	First aid Belluno	23	6 (4.24)	(3 − 9)
	Other	13	7	(7)
Belluno area		**445**		
	Age			
	Adults (45 − 65)	34	1.85 (3.58)	(0 − 13)
	Elderly (> 65)	411	1.31 (3.69)	(0 − 27)
	Sex			
	Men	232	1.17 (3.52)	(0 − 27)
	Women	213	1.55 (3.84)	(0 − 21)
	Access structure			
	First aid Feltre	40	0 (0.00)	(0 − 0)
	First aid Belluno	372	0.93 (3.03)	(0 − 27)
	Other	33	8.5 (6.20)	(1 − 25)

Table 3 describes the mean, minimum, and maximum values of each pollutant concentration at the weather stations of Belluno and Feltre. NO, NO_2_, NOx, and O_3_ concentrations were higher in Belluno, while PM_10_ levels were superior at the Feltre station.

**Table 3:** Summary of statistics for air pollutants from June 2017 to November 2019

Meteorological station	Pollutant or meteorological variable (µg/m^3^)	Minimum	Maximum	Mean ± St dev
Belluno	NO	1.03	43.00	9.62 ± 11.04
	NO_2_	7.06	38.78	17.43 ± 9.27
	NO_X_	10.59	104.70	32.24 ± 25.67
	SO_2_	0.00	2.20	0.54 ± 0.92
	O_3_	8.94	82.47	42.92 ± 23.27
	PM_10_	6.64	33.43	16.12 ± 6.96
Feltre	NO	1.00	21.42	4.11 ± 5.18
	NO_2_	4.74	27.68	11.78 ± 6.66
	NO_X_	5.03	60.68	17.73 ± 14.52
	O_3_	6.77	77.63	37.97 ± 21.29
	PM_10_	8.55	48.00	22.38 ± 12.43
	PM_2.5_	6.23	38.17	17.40 ± 9.50

Table 4 identifies the pollutant concentration and temperature peaks at the Belluno and Feltre stations. December 2017 was pinpointed as a turning point for the pollutants PM_10_, NO_2_, NO, and NO_x_ in Belluno and Feltre while for O_3_ was June 2017 in Belluno and in June 2019 in Feltre.

**Table 4:** Monthly pollutant concentration peaks from June 2017 to November 2019

Pollutant or meteorological variable	Belluno		Feltre	
	Data	Value	Data	Value
NO	December 2017	43.00 µg/m^3^	December 2017	21.42 µg/m^3^
NO_2_	December 2017	38.78 µg/m^3^	December 2017	27.68 µg/m^3^
NO_X_	December 2017	104.70 µg/m^3^	December 2017	60.68 µg/m^3^
O_3_	June 2017	82.47 µg/m^3^	June 2019	77.63 µg/m^3^
PM_10_	December 2017	33.43 µg/m^3^	December 2017	48.00 µg/m^3^
Temperature min	December 2017	− 0.43 ºC	December 2017	-1.64 ºC
Temperature max	June 2017	22.42 ºC	June 2019	23.05 ºC

According to our analysis of the average meteorological values in that period (Table 5), Belluno was windier (0.40 m/s, 169.12° south) than Feltre, while precipitation levels were very similar at both stations. The average temperature was almost similar in Belluno than in Feltre.

**Table 5:** Mean values of additional meteorological variables from June 2017 to November 2019

	Mean wind direction at 5-m vector (degrees)	Mean precipitation (mm)	Mean temperature (ºC)	Mean wind speed (m/s)
Belluno_station_	169.12º (S)	4.87	12.33	0.40
Feltre_station_	162.61º (SSE)	5.30	12.34	0.18

S, south; SSE**,** south, south east

The Spearman rank correlation test was used for hospital admissions, air pollutant concentrations, temperature, and precipitation in Belluno and Feltre areas. A positive correlation was found between hospital admissions and NO, NO_2_, NO_x_, and PM_10_ pollutants in Belluno (0.562, 0.623, 0.568, and 0.665, respectively) as well as in Feltre (0.599, 0.593, 0.595, and 0.614, respectively). Additionally, these admissions had an acceptable correlation (0.635) with pollutant PM_2.5_ and a high inverse correlation with pollutant O_3_ in Belluno and Feltre (− 0.455 and − 0.623, respectively). Regarding the GAMM model for hospital admissions, in the case of Belluno, a statistical association was observed with NO_2_, PM_10_, date and temperature (Table 6). The number of admissions was higher at higher NO_2_ and PM_10_ concentrations. A seasonal pattern of hospital admissions was also observed, with a higher incidence in winter, when temperatures were lower. In the case of Feltre, no variable was significantly associated.

**Table 6:** Fixed effects model in the two study areas: (A) Belluno and (B) Feltre

A) Variables	DF	*P*
S (NO2)	5.155	0.001
S (PM10)	1.000	0.001
S (date)	4.153	0.004
S (temperature)	3.338	< 0.001
B) Variables	*DF*	*P*
S (NO2)	1.000	0.254
S (PM10)	1.000	0.120
S (date)	1.651	0.381
S (temperature)	1.166	0.600

DF, degrees of freedom

Visibility graphs (VGs) were also used to analyze the correspondence or correlation between the monthly concentrations of atmospheric pollutants, mean temperature, and mean precipitation with respect to admissions and accesses in Belluno and Feltre hospitals. Average edge overlap (ω) and interlayer mutual information (IM) were calculated from the MVG built from the time series of the referred variables (Table 7).

**Table 7:** Average edge overlap (ω) and Interlayer mutual information (IM) between hospitalizations and pollutants and meteorological variables from June 1, 2017, to November 30, 2019

	Precipitation	Temperature	NO	NO_2_	NO_x_	O_3_	PM_10_	PM_2.5_
BL								
ω	0.6457	0.7049	0.7206	0.7209	0.7156	0.6805	0.7698	––
IM	0.4920	0.5499	0.4080	0.5585	0.4328	0.4606	0.6292	––
FE								
ω	0.6991	0.6825	0.6772	0.6952	0.6926	0.7168	0.7500	0.7479
IM	0.5020	0.4013	0.4228	0.4879	0.4330	0.4303	0.5468	0.4879

PM_2.5_ was not collected in Belluno

From the combined ω and $IM$ results, the most significant correlations can be seen between hospital admissions and pollutant concentrations of NO_2_ and PM_10_ in Belluno. Such correlations were also evident in Feltre, also including a certain inverse correspondence with O_3_ and a significant correlation with PM_2,5_. In addition, an inverse correspondence exists between the number of hospital admissions and the average temperature in Belluno and between hospital admissions and mean precipitations in Feltre.

**Fig. 2:**
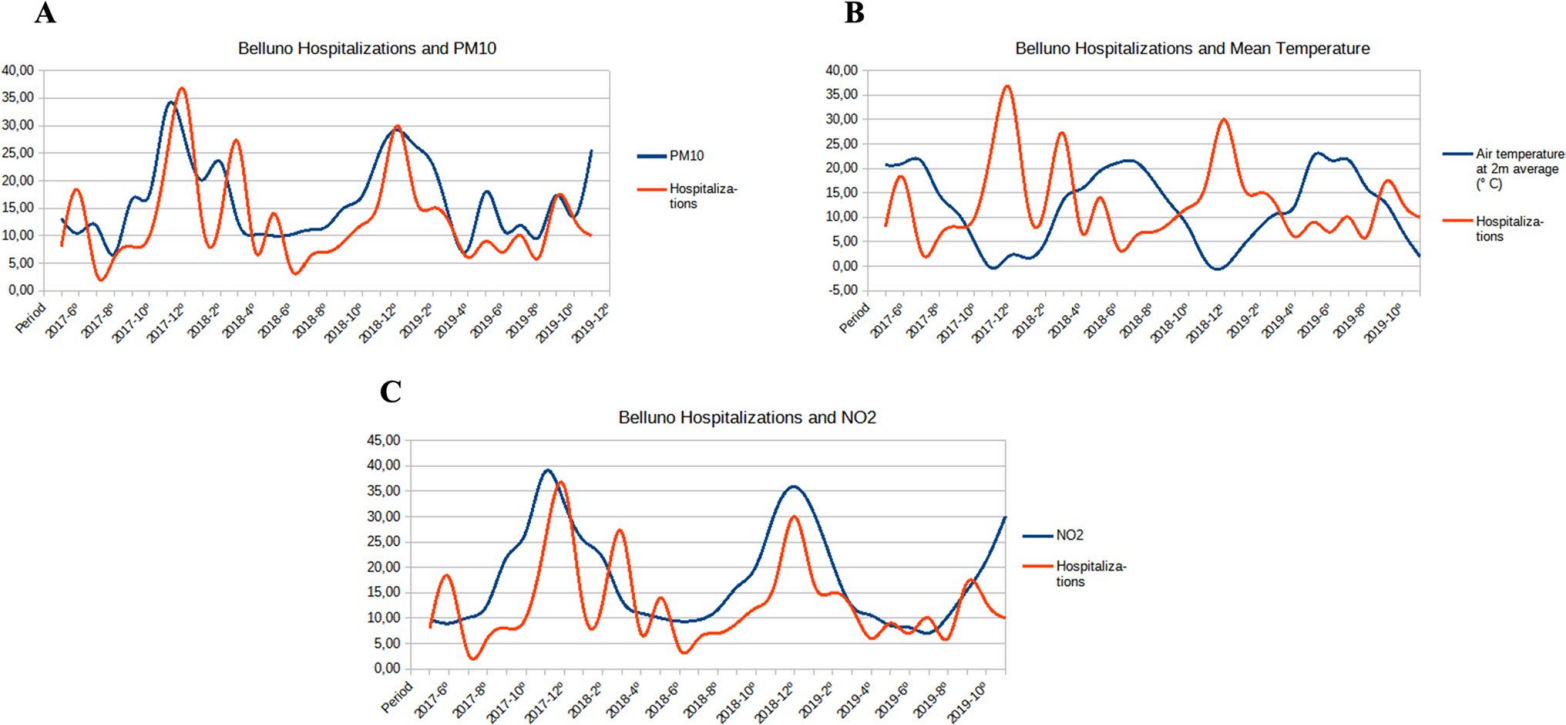
Hospital admissions (red lines) versus **a** PM_10_ concentration, **b** mean temperature, and **c** NO_2_ concentration in Belluno

**Fig. 3:**
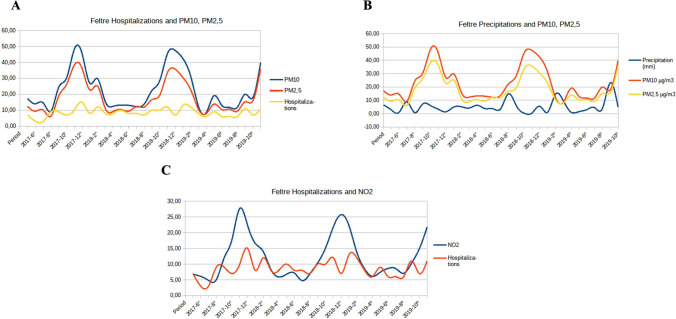
**a** PM_10_ and PM_2,5_ concentrations and hospitalizations, **b** mean precipitations and PM_10_ and PM_2,5_ concentrations, and **c** NO_2_ concentration and hospitalizations in Feltre

We also conducted a graphical comparison between the time series of hospital admissions and some of the pollutant and meteorological variables involved using the available time series data from Belluno and Feltre (Figs. 2 and 3) to visually verify the obtained results. A × 3 factor was applied in the case of mean precipitations and number of hospital admissions to obtain a clearer comparison.

## Discussion

This study, which has been carried out using both the GAMM model and visibility graphs, reveals a significant association between short-term exposure to NO_2_, PM_10_, and PM_2.5_ pollutants and hospital admissions for COPD exacerbations for patients resident in the Belluno and Feltre areas. In addition, by means of Visibility Graphs, it could be also detected that COPD hospital admissions had a certain positive correlation with NO and NO_X_ pollutant concentrations, which behave as a single set, raising the monthly mean of hospital admissions. On the other hand, COPD hospital admissions have been found to increase when mean temperatures are lower in Belluno and when rainfall is lower in Feltre. Such relations are confirmed through the dynamic patterns of the involved variables, showing a marked overlap between NO_2_, PM_2.5_, and PM_10_ values and hospital admissions.

As in other studies (Chang et al. 2020; Priyankara et al. 2021; Jo et al. 2018), we also considered pollutant concentrations, admission date, and environmental temperature in relation to age and sex. Older women had a greater number of hospital admissions when contamination levels increased in autumn. Indeed, hospital admissions were higher for COPD and respiratory illnesses for the older people during the colder periods so that sudden changes in temperature could have a proportional effect on the number of admissions due to an exacerbation of COPD symptoms (Ding et al. 2017; Bao et al. 2020; Huang et al. 2021; Zhu et al. 2020; Lin et al. 2018; Santurtún et al. 2017; Tian et al. 2018).

Each analyzed geographical area showed different results in terms of pollutant levels and hospital admissions, which can be explained by the location of the sensors (city or out-of-town) and the main source of pollution. According to Italian law (legislative decree 155/2010), the annual average limits for the pollutants analyzed are 40 µg/m^3^ for PM_10_, 25 µg/m^3^ for PM_2,5_, 180 µg/m^**3**^ for O_3_, and 40 µg/m^3^ for NO_2_. Therefore, the province of Belluno was well below the permitted Italian limits, but not according to the WHO air quality guideline, where the permitted annual means of PM_10_ and PM_2,5_ are 20 µg/m^3^ and 10 µg/m^3^, respectively, while Feltre was above the limits in both cases (PM_10,_ PM_2,5_) (WHO 2005). The mean values of pollutants were higher in Belluno, except for PM_10_ and PM_2,5_, which was higher in Feltre (Tables 3 and 4).

In both areas, pollutants mainly correlated with hospital admissions were NO_2_, PM_10_, and PM_2.5_ (markedly the latter two) with ω values around or higher than 0.7 and IM values around or higher than 0.5; however, in Belluno, O_3_ had the highest mean concentration levels (42.24 ± 23.33 µg/m^3^), although it does not seem to correlate with hospital admissions. It should be noted that the levels of O_3_ correlated closely with air temperature (0.824) and negatively with some of its precursors and volcanic organic compounds (NO, NO_2_, NO_X_, and PM_10_). In this area, low temperatures also showed a strong correlation with the admissions, with a ω value higher than 0.7 and an IM value of almost 0.55. As regards Feltre, the mean concentration of ozone (37.97 ± 21.29 µg/m^3^) was lower than in Belluno, but it seemed to have a certain correlation with hospitalizations (ω ≈ 0.72, IM ≈ 0.43). There was also a strong correlation between PM_10_, PM_2.5_, and O_3_ (− 0.861, − 0.862, and 0.826, respectively). In addition, the mean concentrations of PM_10_ and PM_2.5_ were higher in Feltre (22.38 ± 12.43 µg/m^3^, 17.40 ± 9.50 µg/m^3^) compared with Belluno. Taking into account the terrain, these values could be explained by the fact that due to the air currents coming from Belluno (168.95° (south), 0.40 m/s), pollutants could accumulate in the bottom of the valley in Feltre. Indeed, home heating systems constitute a significant source of pollution levels in the province, and the pollutants generated by these heating systems presented peaks in winter which followed the fluctuations in environmental temperature. Also, Krachunov et al. (2017) reported that the concentrations of NO_2_ and O_3_ could be linked through chemical reactions. Authors explained that much of the NO_2_ measured in cities are originated from secondary production through reaction of NO with O_3_ (generating NO_2_ and removing O_3_).

As mentioned above, our findings could be explained by the geographical and cultural characteristics of the province of Belluno, and this possible association has been put forward in other works. Chen et al. (2004) found a positive correlation between low PM_10_ and PM_2.5_ levels and admissions for COPD of older people over 65 years old in Vancouver (Canada). Yang et al. (2005), also in Vancouver, reported very similar results linking climate with pollution, but unlike our study, the main predictor was NO_2_ and not the PM_10_ level.

Another factor to be taken into account is the exposure time. In studies carried out in Demark and Sweden to analyze long-term exposure to low levels of pollutants in middle-aged and older adults, the levels of PM_2.5_ and NO_2_ were positively associated with COPD incidence. Here, the values of NO_2_ and O_3_ levels were higher compared with our study, although not PM_10_ (Liu et al. 2021). Regarding the short-term impact, De Vries et al. (2017), in a study where the level of pollutants and average temperature was very similar to our study, found strong positive associations between outdoor SO_2_ and NO_2_ and COPD exacerbation.

In our study, a seasonal effect was observed, with most hospital admissions for COPD occurring in the autumn or winter months. In fact, as shown in Figs. 2 and 3, lower temperatures and higher concentrations of pollutant levels occurred in these colder seasons. Similar results were observed by Krachunov et al. (2017), who significantly associated levels of air pollutants with lower daily mean temperatures. In this context, Lin et al. (2018) identified temperature as a potential risk factor for COPD, since an increase in temperature in spring and a decrease in temperature in autumn were both associated with an increased risk of COPD hospitalization. They concluded that air pollution with increased NO_2_, CO, O_3_, and PM_10_ concentrations and continual temperature changes were associated with acute exacerbation of COPD in older patients.

In the present study, several limitations should be considered. Firstly, we observed environmental agents at the monitoring stations but not at the patients’ homes. In fact, we considered the environmental agents obtained at the monitoring stations closest to the place of residence. Secondly, the COVID-19 pandemic occurred in 2020, at the end of our study period. Hospital admissions were certainly reduced in pandemic period due to the increased demand generated by COVID-19, when only patients with severe pathologies were admitted. Moreover, pollution levels could have been altered by the lockdown, when many workplaces closed. Therefore, although we have meteorological and hospital admission data up to June 2020, it was considered not to include these data. Thirdly, the data were collected from institutional databases, so in some cases, they were incorrectly recorded by the health professional and have not been considered for the present study. Finally, we were unable to use time lags and test time exposure. Despite this, the innovative analysis we used for this (with multiplex visibility graphs) allowed us to obtain an overlay of several variables at the same time.

## Conclusions

Our results showed a significant association between short-term exposure to concentrations of PM_10_ and hospital admissions for COPD in the Belluno province. Other environmental agents have also been associated with hospital admissions in this area. In addition, climatological and geographical aspects could be related with air pollution and, as a result, with hospital admissions associated to COPD. The multiplex visibility graph approach, as with the models already used in this area (GAMMs), is an innovative time series tool that is very useful for understanding the overlap between variables.

Our results provide evidence for the impact of environmental pollution levels on the health system. Therefore, for health planning purposes, these data could be taken into account in order to improve health care services for COPD.

## Data Availability

The datasets generated during and/or analyzed during the current study are available from the corresponding author on reasonable request.
